# Immune profiling of gastric adenocarcinomas in EU and LATAM countries identifies global differences in immune subgroups and microbiome influence

**DOI:** 10.1038/s41416-025-02979-6

**Published:** 2025-03-20

**Authors:** Tessa S. Groen – van Schooten, Manuel Cabeza-Segura, Rui M. Ferreira, Carolina Martínez-Ciarpaglini, Rita Barros, João Santos-Antunes, Andreia Costa, Edith A. Fernández-Figueroa, Leonardo Lino-Silva, Angélica Ixtaccihuatl Hernandez-Guerrero, Erika Ruiz-García, Carmelo Caballero, Hugo Boggino, Cinthia Gauna, Daniel Cantero, Berenice Freile, Federico Esteso, Juan O´Connor, Arnoldo Riquelme, Gareth Owen, Erick Riquelme, Juan Carlos Roa, Gonzalo Latorre, Marcelo Garrido, Fiorella Ruiz-Pace, Marc Diez García, Maria Alsina, Florian Lordick, Judith Farrés, Juan Antonio Carbonell-Asins, Rossana Villagrasa, Rita Pereira, Roos E. Pouw, Elena Jimenez-Martí, Ana Miralles, Rodrigo Dientsmann, Ceu Figueiredo, Fatima Carneiro, Andrés Cervantes, Sarah Derks, Tania Fleitas

**Affiliations:** 1 Department of Medical Oncology, Amsterdam University Medical Center (UMC) location Vrije Universiteit Amsterdam, Amsterdam, Netherlands; 2 Cancer Biology and Immunology, Cancer Center Amsterdam, Amsterdam, Netherlands; 3 Oncode Institute, Amsterdam, Netherlands; 4 Department of Medical Oncology, Hospital Clinico Universitario, INCLIVA, Biomedical Research Institute, University of Valencia, Valencia, Spain; 5 i3S - Instituto de Investigação e Inovação em Saúde, Universidade do Porto, Porto, Portugal; 6 Ipatimup - Institute of Molecular Pathology and Immunology of the University of Porto, Porto, Portugal; 7 Pathology Department. Hospital Clínico Universitario de Valencia, INCLIVA, Valencia, Spain; 8 Faculty of Medicine of the University of Porto, Porto, Portugal; 9 Department of Pathology, Unidade Local de Saúde São João, Porto, Portugal; 10 Department of Gastroenterology, Unidade Local de Saúde São João, Porto, Portugal; 11 Department of Oncology, Unidade Local de Saúde São João, Porto, Portugal; 12 Núcleo B de Innovación en Medicina de Precisión, Instituto Nacional de Medicina Genómica, Ciudad de, México, México; 13 Head of Division. Surgical Pathology, National Cancer Institute (INCan), Mexico City, Mexico; 14 Department of Gastrointestinal Endoscopy, Instituto Nacional de Cancerología, Mexico City, Mexico; 15 Departamento de Tumores de Tubo Digestivo, Instituto Nacional de Cancerología, Ciudad de, México, México; 16 Laboratorio de Medicina Traslacional, Instituto Nacional de Cancerología, Ciudad de México, México; 17 Department of Pathology, GENPAT, Asunción, Paraguay; 18 Medical Oncology Department, Instituto de Previsión Social, Asunción, Paraguay; 19 Department of Gastroenterology, Instituto de Previsión Social, Asunción, Paraguay; 20 Medical Oncology Department, Instituto Alexander Fleming, Buenos Aires, Argentina; 21 Department of Gastroenterology, Faculty of Medicine. Pontificia Universidad Catolica de Chile. Center for Prevention and Control of Cancer (CECAN), Santiago, Chile; 22 Faculty of Biological Sciences & Faculty of Medicine. Pontificia Universidad Católica de Chile, Millennium Institute for Immunology and Immunotherapy, Center for Prevention and Control of Cancer (CECAN), Advance Center for Chronic Disease (ACCDIS), Santiago, Chile; 23 Department of Respiratory Diseases, Faculty of Medicine. Pontificia Universidad Católica de Chile, Santiago, Chile; 24 Department of Pathology, Faculty of Medicine. Pontificia Universidad Católica de Chile, Santiago, Chile; 25 Department of Gastroenterology, Faculty of Medicine. Pontificia Universidad Catolica de Chile, Santiago, Chile; 26 Facultad de Ciencia de la Salud, Centro de Oncología de Precision, Universidad Mayor, Huechuraba, Chile; 27 Oncology Data Science, Vall d’Hebron Institute of Oncology, Barcelona, Spain; 28 Medical Oncology Department, Vall d’Hebron Institute of Oncology, Barcelona, Spain; 29 Hospital Universitario de Navarra, Navarrabiomed-IdiSNA, Pamplona, Spain; 30 Department of Medicine (Oncology, Gastroenterology, Hepatology, and Pulmonology), University of Leipzig Medical Center, Comprehensive Cancer Center Central Germany (CCCG), Leipzig, Germany; 31 Anaxomics Biotech, S.L., Barcelona, Spain; 32 Department of Bioestatistics, INCLIVA Biomedical Research Institute, Valencia, Spain; 33 Department of Gastroenterology, Hospital Clínico Universitario de Valencia, Valencia, Spain; 34 Gastroenterology Department. Amsterdam UMC, Amsterdam, The Netherlands; 35 CiberOnc. Carlos III Institute, Madrid, Spain

**Keywords:** Translational research, Cancer, Biomarkers

## Abstract

**Background:**

Gastric cancer (GC) patients from European (EU) and especially Latin American (LATAM) countries are underrepresented in previous large-scale multi-omic studies that have identified clinically relevant subgroups. The LEGACY study aimed to profile the molecular and immunological features of GCs from EU and LATAM countries.

**Methods:**

Tumor biopsies from 95 EU and 56 LATAM GCs were profiled with immunohistochemistry (CD3, CD8, FOXP3, PD-L1, MSI and HER2), Nanostring mRNA expression analyses, and microbiome sequencing.

**Results:**

Immune profiling identified four distinct immune clusters: a T cell dominant cluster with enriched activation pathways, a macrophage dominant cluster and an immune excluded microenvironment which were equally distributed among the countries. A fourth cluster of mostly Mexican patients consisted of excessive T cell numbers accompanied by enhanced cytokine signaling in absence of enhanced antigen presentation and cytotoxicity signatures and a strong association with *H. pylori* infection.

**Discussion:**

Both EU and LATAM countries have GCs with a T cell inflamed microenvironment that might benefit from checkpoint inhibition. We identified a highly inflamed GC subgroup that lacked antigen presentation and cytotoxicity associated with *H. pylori* CagA-positive strains, suggesting their contribution to tumor immune tolerance. Future studies are needed to unravel whether these cancers benefit from immunotherapy as well.

## Introduction

Gastric cancer (GC) is a deadly disease leading to 659,853 annual deaths worldwide in 2022 [1]. The incidence of this disease varies across the world, with the highest age-standardized rates in Asia, followed by Latin America (LATAM), the Caribbean, Europe, and being lowest in Africa [2]. Geographic diversity in GC is not fully understood but is likely associated with differences in the prevalence of risk factors such as persistent *Helicobacter pylori* infection, genetic predisposition, and lifestyle-related factors [3]. Recent data also point to a link between the microbiota and GC, but the extent to which the microbiota may explain GC geographic differences is unclear [4, 5].

Advanced stage GC has a poor prognosis with a 5-year survival of <5% in case of metastatic disease as systemic therapies are only limited effective [6]. The standard of care for this setting includes chemotherapy with a fluoropyrimidine-platin doublet and, in case of HER2 overexpression additional trastuzumab, which improves median survival from 11 to 16 months [7, 8]. Immune checkpoint inhibitors (ICI) are a recent addition to chemotherapy for HER2-negative and HER2-positive disease and greater improvements in case of PD-L1 positivity [9–12]. Although ICI improves median overall survival from 11.4 months to 14.1 months in the CheckMate 649 study, responses vary greatly between patients, likely as a result of differences in anti-tumor immune activity [10]. In previous studies we have observed that the tumor immunity depends on molecular subtype. GCs with microsatellite instability (MSI) or positivity for the Epstein Barr Virus (EBV) are highly T cell inflamed which was shown to be associated with durable responses to ICI. Moreover, not only the T cell infiltrates but also the immune signaling impacts the response to ICI [13].

Besides molecular subtypes, geographic location is associated with specific immune features. Different transcriptome studies have shown that tumors from Asian patients contain fewer CD8 T cell markers compared to cancers from non-Asian countries [14]. Furthermore, GCs of patients from so-called Western countries (Australia, North-America and Western-Europe) had higher CD4+ and CD8+ T cell scores compared to GCs of patients from Asian and Brazilian descents [15]. Geographic differences are also observed in responses to ICIs. For example, in lung cancer and other cancer types, patients from Asia benefit more from ICI compared to non-Asian populations, although this is not clear for GC [16]. Patients from Europe and particularly LATAM are underrepresented in global clinical studies, and it is not known how patients from these continents respond to ICI [10].

To unravel the molecular and immunological features of GC from EU and LATAM specifically, the LEGACY consortium profiled GCs from EU and LATAM countries in relation to the cancer genome, immunome, microbiota, histopathological features, and epidemiology [17].

In the present study, we aimed to characterize the immune landscape of advanced GC from patients from EU and LATAM countries. This will not only improve our biological understanding of the disease but can also inspire the development of a global immune targeting strategy.

## Methods

### Patient material

Patient material and patient characteristics, including disease outcomes were collected as part of an Ethical Review Board approved trial called LEGACy (NCT04015466) [17] and CEl/1412/19 from INCAN. More details on the ethical approvals are described in the Ethical Approvals section. As part of a sizeable standardized tissue sampling effort tumor biopsies and resection material were prospectively collected from 293 patients with primary, treatment-naive advanced gastric adenocarcinoma (GC) including tumors of the gastro-esophageal junction (AEG II and AEG III according to Siewert and Stein) [18]. Patients were recruited between 2019 and 2022 in the participating hospitals from four hospitals in three EU countries (Spain, Portugal, and the Netherlands) and four hospitals from LATAM countries (Argentina, Mexico, Chile, and Paraguay). Data collection and handling of patient material has been standardized through a consensual lab handbook. Biopsies from the tumor and adjacent normal tissue are formalin-fixed and paraffin-embedded (FFPE) at each site and shipped to Ipatimup (Portugal) for pathological examination and immunohistochemistry, and then sent to VHIO, Spain for DNA analysis and to Amsterdam UMC, Netherlands for mRNA analysis. Fresh frozen samples from biopsies from tumor-adjacent normal gastric mucosa were collected in parallel and centrally processed at Ipatimup (Portugal) for microbiota analysis.

### Pathological review

Histology was assessed by an expert pathologist on H&E stains, and tumors were classified according to both Lauren’s classification and the 5th edition of the World Health Organization Classification of Tumors of the Digestive System. Tumor areas from all biopsies were annotated before further processing.

### Immunohistochemistry

FFPE samples were cut in 3 μm sections and stained with Ventana® BenchMark ULTRA (Roche) system together with the OptiView DAB IHC Detection Kit (#760-700, Roche) following standard protocol for the following proteins: PD-L1 (Anti-PDL1 Clone 22C3, Dako), CD3 (NCL-L-CD3-565 Clone LN10, Leica), CD8 (#790-4460 Clone SP57, Roche), FoxP3 (#12653 Clone D6O8R, Cell Signaling), MSH2 (#790-5093 Clone G219-1129, Roche), MSH6 (#790-5092 Clone SP93, Roche), MLH1 (#790-5091 Clone M1, Roche) and HER2 (#790-2991 Clone 4B5, Roche). For all antibodies antigen retrieval is performed with CC1 (EDTA) and hematoxylin is incubated for 8 min. For each slide to be stained, a positively staining control tissue was added. EBV status and HER2 status were determined using in situ hybridization for detecting HER2 amplification (Dual ISH DNA Probe Cocktail, 800-4422, Roche) and Epstein-Barr Virus Early RNA (EBER probe, 800-2842, Roche) with the Ventana® system, together with the ultraView SISH DNP Detection Kit (#800-098, Roche) and ultraView Red ISH DIG Detection Kit (#800-505, Roche).

All samples were reviewed by two expert pathologist (FC and CMC) and the expression of mismatch repair proteins, HER2 and EBER was classified according to standard criteria [19, 20]. Positive lymphocytes for immune markers were counted using the positive cell detection script of the open-source software Qupath [21]. For CD3 and CD8 two hotspot areas of 0.30 mm^2^ were selected. For FOXP3 counting, one hotspot area of 0.20 mm^2^ was selected. Normal mucosa, gastritis, granulation tissue, and necrosis were avoided. The combined positive score (CPS) for PD-L1 was evaluated following the PD-L1 IHC 22C3 pharmDx Interpretation Manual for gastric cancer (Interpretation manual Dako. The number of positive mononuclear inflammatory cells (cytoplasmic or membranous staining) and positive tumor cells (presenting membranous staining) were recorded separately.

### Nanostring mRNA expression analyses

Annotated tumor areas were manually cut out of 10 μm FFPE slides using a scalpel. RNA was isolated from the FFPE tumor material using the Qiagen RNeasy FFPE kit (Qiagen, Germantown, MD). RNA quantity was measured by Qubit RNA IQ Assay on the Qubit 4 fluorometer (Thermo Fisher, US). The RNA quality was measured with an Agilent RNA 6000 Nano kit using Bioanalyzer instrument (Agilent Technologies, US). mRNA was analyzed using the NanoString nCounter® analysis system with the Nanostring PanCancer Immune Profiling panel (NanoString Technologies, Seattle, WA). Data was analyzed using the Nanostring nSolver software version 4.0. All samples used for the final analysis have passed quality control for quality control of imaging, binding density, positive linearity and limit of detection and the data has been normalized according to housekeeping genes. For advanced analysis, encompassing cell type and pathways scores as well as differential expression, the nCounter Advanced analysis 2.2 plugin was used and the accompanying nanostring celltype and pathway annotations were utilized.

### Microbiota analysis

DNA isolation from fresh frozen biopsies from tumor-adjacent normal mucosa was performed using the QIAamp DNA FFPE Tissue Kit (QIAGEN) according to manufacturer’s instructions [4]. The 16S rRNA gene was amplified at the V5-V6 hypervariable region. The sequencing library was sequenced in an Illumina MiSeq platform (Illumina, San Diego, CA, USA), with a read length of 300 bp paired-end reads and an expected output of 100,000 reads per sample [22]. Reads were then quality filtered by imposing a maximum number of expected errors of 1.0 and trimmed at a fixed length. Filtered reads were dereplicated and amplicon sequence variants (ASVs) were clustered using the UNOISE algorithm [23]. Next, each biological sequence was taxonomically assigned using SINTAX algorithm [24] with the 16S RDP Classifier v16 training set as the reference database. Sequence data analysis was performed using usearch_v8.1.11861_i86linux64 and usearch11.0.667_i86linux32 [25].

### Statistical analysis

IBM SPSS Statistics version 26.0 (IBM Corporation, Armonk, NY, USA), R4.4.0 and Graphpad prism were used for performing statistical analysis and making corresponding figures. To compare three groups, Kruskal–Wallis test with Dunn’s post correction was used. To compare two groups, normality test was first run to determine distribution and Mann–Whitney U test was used. Statistical analysis of differential gene expression data, both at gene and pathway levels, was performed using nCounter Advanced Analysis Software 2.0 (NanoString Technologies). *p*-value was adjusted for multiple comparisons using the false discovery rate proposed by Benjamini and Hochberg [26].

## Results

### Gastric cancers from LATAM are less often MSI and express less PD-L1

**Fig. 1:**
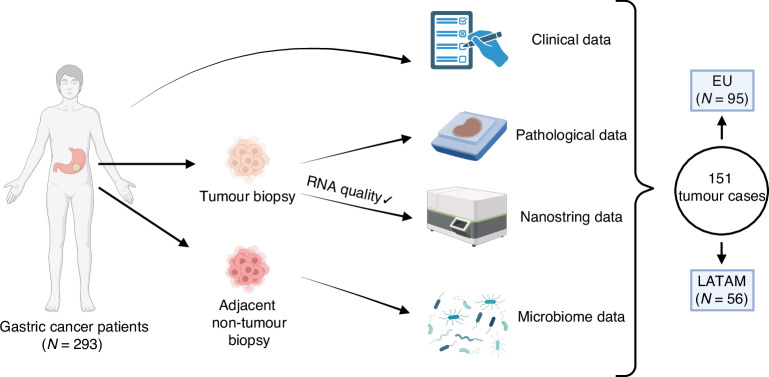
Study outline. EU Europe, LATAM Latin America.

In this prospective study, we included tissue biopsies from 293 advanced stage GCs as well as 113 fresh-frozen biopsies from non-cancer adjacent tissue to analyze the microbiota. After shipment and extraction of slides for pathological and DNA analyses, 238 samples were available for RNA extraction. Eighty-seven samples were excluded due to insufficient tumor material or low RNA quality leading to a total number of 151 samples with sufficient quantity and quality RNA. Of these, 63% (*n* = 95) were from EU countries [Portugal (*n* = 17), Spain (*n* = 37), and The Netherlands (*n* = 41)], and 37% ((*n* = 56)) were from LATAM countries [Argentina (*n* = 7), Chile (*n* = 18), Paraguay (*n* = 15) and Mexico (*n* = 16) (Fig. 1).

The clinicopathological details per continent are listed in Table 1. According to Lauren’s classification, the most common tumors were of the intestinal type (88/151, 58%) and 90 (59%) of the patients were male. In 96 out of 151 cases (64%), the tumors had spread to distant organs. Among the tumors, 18 (12%) were MSI and 6 (4%) were EBV-positive. Additionally, more than half of the tumors had a PD-L1 CPS ≥ 5 (90/151, 60%).

**Table 1:** Clinicopathological characteristics of the study population.

	Total		EU		LATAM		*p-value*
Characteristic	*N*	%	*N*	%	*N*	%	
Patients enrolled	151	100	95	63	56	37	
Age (median, range, Y)	67	26–89	67	31–89	66	26–83	0.132
Gender							0.466
Male	90	60	54	57	36	64	
Female	61	40	41	43	20	36	
Race							**<0.001**
White Hispanic or Latino	70	46	18	20	52	95	
White not-Hispanic nor Latino	69	46	66	73	3	5	
Asian	1	1	1	1	0	0	
Black or African American	6	4	6	7	0	0	
Tumor Location							**0.046**
GE Junction/Cardia/Proximal	45	30	29	31	16	29	
Fundus/Body	60	40	32	34	28	50	
Antrum/Distal	42	28	33	35	9	16	
Lauren’s classification							0.174
Intestinal	88	58	59	62	29	53	
Diffuse	51	34	27	28	24	44	
Mixed	10	7	8	8	2	4	
Medullary	1	1	1	1	0	0	
cTNM staging							
Primary tumor (T)							**<0.001**
1	2	1	2	2	0	0	
2	6	4	6	6	0	0	
3	47	31	41	43	6	11	
4	56	37	31	33	25	45	
x	40	26	15	16	25	45	
Regional lymph nodes (N)							**<0.001**
0	9	6	8	8	1	2	
1	25	17	20	21	5	9	
2	28	19	26	27	2	4	
3	25	17	15	16	10	18	
x	64	42	26	27	38	68	
Distant metastasis (M)							0.086
0	55	36	40	42	15	27	
1	96	64	55	58	41	73	
Molecular characterisation							**0.028**
MSI/EBV−	18	12	16	17	2	4	
MSS/EBV−	126	83	74	79	52	93	
MSS/EBV+	6	4	4	4	2	4	
HER2 status							0.467
Negative	136	90	83	90	53	95	
Positive	12	8	9	10	3	5	
CPS score							**0.002**
0–5%	52	34	22	23	30	54	
5–10%	14	9	11	12	3	5	
>10%	76	50	54	57	22	39	
IHC (mean, range)							
CD3	3797	37–20,083	4104	473–20,083	3289	37–9103	0.124
CD8	2718	17–23,563	2874	17–23,563	2453	107–8593	0.382
FOXP3	556	35–2820	616	35–2820	457	35–1440	0.056

*P*-values ≤0.05 were considered statistically significant (in bold).

*CPS* combined positive score, CD3 CD8 and FoxP3 given in counts per mm^2^, *ns* non-significant, *EBV* Epstein Barr Virus, *MSI* microsatellite instable, *MSS* microsatellite stable, *HER2* Human Epidermal growth factor Receptor 2, *IHC* Immunohistochemistry, *EU* Europe, *LATAM* Latin America.

When comparing patients from EU and LATAM, we observed that EU patients had more antrum-distal located tumors (35% vs 16%, *p* = 0.046), while tumors from patients of LATAM countries were mostly located at the fundus/body (50%). Furthermore, MSI was more often detected in the EU cohort (17% vs 4%, *p* = *0*.028) and also PD-L1 CPS ≥ 5 (65/95, 68%) was more often detected in EU tumors compared to LATAM tumors (25/56, 45%, *p* = *0*.002) (Table 1). Clinicopathological differences were also analyzed per country which showed that patients from Argentina and Mexico are significantly younger (*p* = 0.006). A higher proportion of patients with distant metastases was also observed in patients from Spain, Chile and Mexico (*p* < 0.001). Moreover, although no significant differences were observed in the proportion of MSI or EBV-positive patients between countries (*p* = 0.618), patients from Chile and the Netherlands had the highest frequencies of PD-L1 CPS > 5 (add percentage *p* < 0.001) (Supplementary Table 1).

We next evaluated the immune infiltrate of advanced GCs using immunohistochemistry (IHC) with antibodies against CD3, CD8, and FoxP3. Overall, intratumoral CD3-positive cells varied in number from 37 to 20,083 counts per mm^2^ (mean 3797), CD8-positive cells ranged from 17 to 23,563 counts per mm^2^ (mean 2718), and FoxP3-positive cells ranged from 35 to 2820 counts per mm^2^ (mean 556). As expected, MSI and EBV-positive tumors had more CD8+ T cells compared to MSS/EBV-negative tumors. Besides a trend towards less FOXP3-positive cells in LATAM cancers no differences in number of CD3^+^ or CD8^+^ cells were observed (Table 1, Supplementary Table 1).

### Transcriptomic analysis identified distinct GC immune clusters

**Fig. 2:**
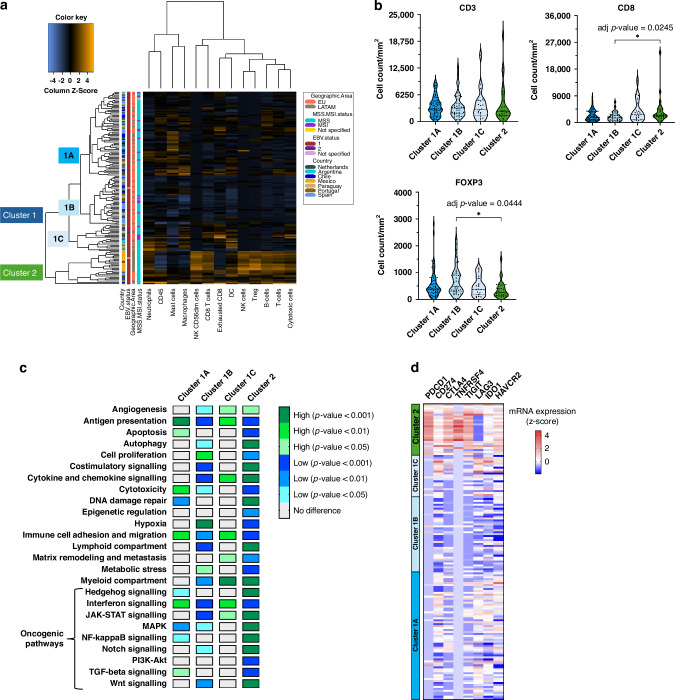
Transcriptomic analysis identified distinct GC immune clusters. **A** Unsupervised hierarchical clustering heatmap based on transcriptional cell type scores identifying two main GC immune clusters: cluster 1 (subdivided into clusters 1A, 1B and 1C) and cluster 2. **B** IHC data validating the four immune scenarios detected by the transcriptomic analysis. Adj *p* < *0.05* were considered as statistically significant. **C** Differential pathways expression across the four distinct immune clusters. *P* < 0.05 were considered as statistically significant. **D** Heatmap showing the z-score mRNA expression of gene targets for ICIs and new immunotherapies: *PDCD1*, *CD274*, *CTLA4*, *TNFRSF4*, *LAG3*, *IDO1*, and *HAVCR2*. EU Europe, LATAM Latin America.

We next used Nanostring mRNA expression data to further characterize immune features of both cohorts. We first performed an unsupervised hierarchical clustering based on cell type scores and identified two main GC immune clusters: cluster 1 (*n* = 125 patients) and cluster 2 (*n* = 26 patients) (Fig. 2A). Cluster 2 was characterized by higher scores for B cells, CD8 T cells, cytotoxic cells, dendritic cells, neutrophils, NK cells, T cells, and T regulatory cells (all *p* < 0.0001; Supplementary Fig. 1A). Cluster 1 contained tumors with a more heterogeneous immune infiltration and could be subdivided by three subclusters: cluster 1A (*n* = 66 patients), characterized by an enrichment of mast cells, B cells, and exhausted CD8 T cells; cluster 1B (*n* = 38 patients), characterized by very low numbers of immune cells; and cluster 1C (*n* = 21 patients), with an immune infiltrate dominated by macrophages and dendritic cells. Validation of these four immune scenarios with IHC data confirmed significantly higher CD8+ cell counts (adj *p* = 0.0245) and lower FOXP3-positive cells in cluster 2 compared to cluster 1B (adj *p* = 0.0444, Fig. 2B).

We next looked at the difference in immune pathway expression between the clusters. Interestingly, while having generally lower immune cell scores in cluster 1A compared to cluster 2, cluster 1A was enriched for antigen presentation, cytotoxicity, and immune cell adhesion and migration compared to the other clusters (adj *p* < 0.05, Fig. 2C). Cluster 1B showed higher expression of hypoxia and low number of immune cells and cluster 1C was enriched for extracellular matrix remodeling (adj *p* < 0.05, Fig. 2C).

At last, we determined oncogenic pathway signaling for all four clusters and identified that highly inflamed cluster 2 was enriched for Hedgehog, JAK-STAT, MAPK, NF-κB, Notch, and Wnt signaling pathways (adj *p* < 0.05, Fig. 2C), and presented a downregulation of interferon, PI3K-Akt, and TGF-β networks (adj *p* < 0.05, Fig. 2C), in comparison with the other clusters. Cluster 1B had significantly lower scores for JAK-STAT, MAPK, interferon, and Wnt signaling pathways in comparison with the other subclusters in cluster 1 (adj *p* < 0.05, Fig. 2C). Finally, analysis of the inter-cluster variability in gene expression of targets for ICIs and new immunotherapies showed that patients belonging to cluster 2 presented higher expression of immune checkpoints, such as *PDCD1*, *CD274* (encoding PD-L1), *CTLA4*, *TNFRSF4* (encoding OX40), *TIGIT* and *HAVCR2* (encoding TIM3), compared to clusters 1A, 1B, and 1C (Fig. 2D, Supplementary Fig. 1B) likely indicative for the high number of T cells in this cluster. The clusters did not differ in MSI or HER2 status (Supplementary Table 2).

### Gastric cancer immune profiles show geographic specificities

**Fig. 3:**
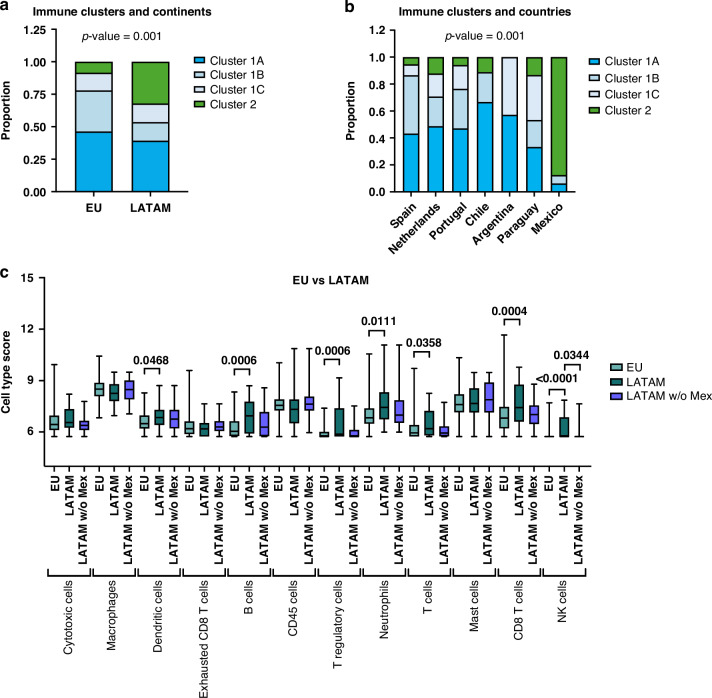
Gastric cancer immune profiles show geographic specificities. **A** Distribution of the four immune cluster showed differences between EU and LATAM continents (*p* = 0.001). **B** Distribution of the four immune clusters showed differences across countries (*p* = 0.001). **C** Immune cell type abundance comparison between EU and LATAM GCs patients with and without Mexico. EU Europe, LATAM Latin America, w/o Mex without Mexican population.

We next evaluated the geographic differences across the four immune clusters and observed that the distribution of the immune clusters between EU and LATAM GCs was significantly different (*p* = 0.001, Fig. 3A). Cancers from the EU were more often in the non-inflamed cluster 1B (32% vs. 14% in LATAM), while cluster 2 was overrepresented in LATAM (32% vs. 8% in EU). Within the EU-group of countries not many differences were observed except for an overrepresentation of cluster 1B in GCs from Spain (Fig. 3B). Within LATAM, more country specificity was observed. In particular, 88% of GCs from Mexico were represented in the highly inflamed cluster 2, while immune-poor cluster 1B did not contain any Argentinian GC patients (Fig. 3B).

In analogy of immune cluster associations, cancers from Mexico showed the highest number of dendritic cells (*adj p* = 0.0468), B cells (*adj p* = 0.0006), T regulatory cells (*adj p* = 0.0006), neutrophils (*adj p* = 0.0111), CD8 T cells (*adj p* = 0.0004) and NK-cells (*adj p* < 0.0001) (Fig. 3C). Without tumors from Mexico, no differences in immune composition were observed between tumors from EU and LATAM, indicating that in both continents GC can have different levels of antitumor immunity.

Regarding the patients belonging to cluster 2, we observed that the lack of cytotoxicity and antigen presentation was mainly related to the Mexican population (Supplementary Fig. 2A). Although the Mexican patients presented high number of CD8-positive T cells, they also were the ones with the highest number of T regulatory cells, which may play a role in dampening the cytotoxic immune response (Supplementary Fig. 2B). Furthermore, these T cells also showed high expression of immunomodulatory genes such as *CTLA4*, *TNFRSF4*, *TIGIT* and *HAVCR2* (Supplementary Fig. 2C).

### Helicobacter sp. and Lactobacillus are more abundant in GC immune cluster 2

**Fig. 4:**
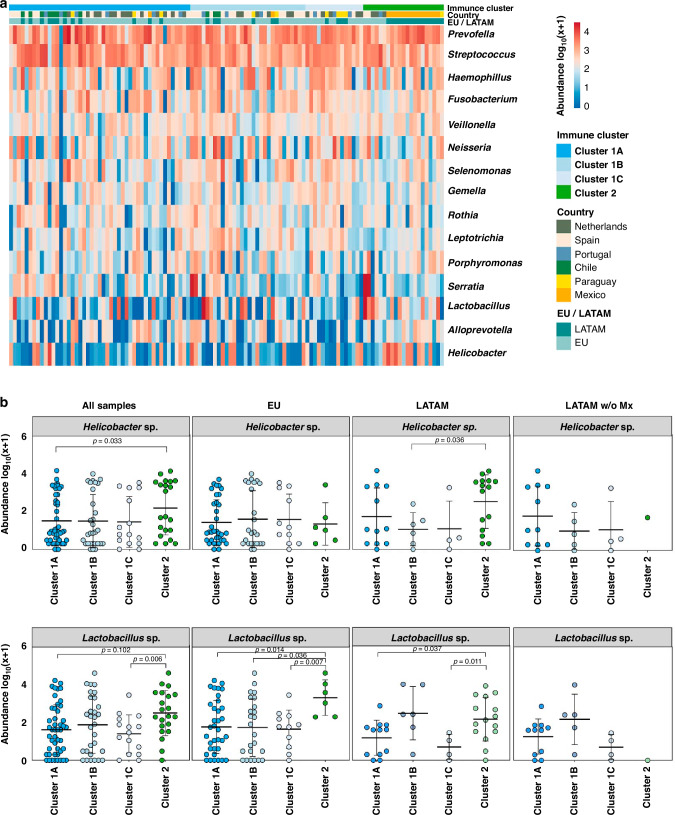
Relationship between the microbiota and the immune profiling of gastric cancer patients. **A** Heatmap showing a supervised clustering analysis of GCs based on the top 15 most abundant genera. **B** Abundance of Helicobacter sp. and Lactobacillus sp. between immune clusters in all cases, per EU and LATAM GCs, and in LATAM excluding Mexican GCs.

As differences in the microbiome can provide an explanation for geographical immune differences, we next analyzed the microbiota in 113 (75%) of the 151 tumors from which fresh frozen tissue was available. The analyses showed that the most abundant bacteria found in the stomach of GC patients included the genera *Prevotella*, *Streptococcus*, *Haemophilus*, *Fusobacterium*, *Veillonella*, *Neisseria*, *Lactobacillus*, and *Helicobacter*, among others, without significant differences observed between EU and LATAM GCs (Supplementary Fig. 3). Overall, there was no significant clustering between the microbiota and specific immune clusters (Fig. 4A). However, *Helicobacter* sp. was significantly more abundant in cluster 2 than in cluster 1A (Fig. 4B). The same accounted for *Lactobacillus* sp. which was significantly more abundant in cluster 2 compared to clusters 1A and 1C (Fig. 4B), with no additional statistically significant differences observed for the other genera (Supplementary Fig. 4). We next assessed the abundances of these taxa in each cluster by continent and identified that in LATAM GCs *Helicobacter* sp. was significantly more abundant in cluster 2 than in cluster 1B, but this difference was not observed in EU GCs (Fig. 4B). The prevalence of the virulence marker CagA was higher in LATAM (67%) than in EU (33%) *H. pylori* strains, but without statistical significance (*p* = 0.33).

We next explored the relationships between the *H. pylori* CagA status and the immune characteristics of cluster 2. Patients infected with CagA-positive strains had increased (non-significant) number of T regulatory cells, and significantly decreased cytotoxicity, number of macrophages, and antigen presentation signature (Supplementary Fig. 5), suggesting that these strains contribute to tumor immune evasion. In EU GC patients *Lactobacillus* sp. was significantly more abundant in immune cluster 2 than in all other clusters, while in LATAM GCs this increased abundance was only observed in comparison with clusters 1A and 1C (Fig. 4B). When the analysis was performed by removing the samples from Mexico, the relationships between *Helicobacter* sp. and *Lactobacillus* sp. cluster 2 were not maintained, suggesting that at least the association between *Helicobacter* sp. and a T cell inflamed microenvironment was Mexico specific.

## Discussion

In this study of the LEGACY consortium, we had the unique opportunity to profile the immune microenvironment of advanced GCs from European and Latin American patients, with standardized methods to sample tumor and adjacent normal tissue from patients in seven different countries. We identified four distinct immune clusters with different levels of immune activation. While all countries had tumors with more and less abundant immune infiltrates, cancers from Mexico had an immune microenvironment with an exceptional immune composition that clustered separately from all other cancers. Microbiota analyses identified a strong association of these cancers with *Helicobacter* sp. infection. Additional immune pathway analyses identified that these highly immune inflamed cancers were limited in expression of pathways associated with antigen presentation and cytotoxicity, while high in expression of pathways associated with DNA repair, NF-κB, MAPK signaling, and autophagy. As *H. pylori* is known to induce DNA damage and activate MAPKs and transcription factors NF-κB and AP-1, high abundance of the bacterium in these CGs may support such inflamed immune profile [27, 28]. The lack of antigen presentation and cytotoxicity argues in favor of an anti-microbiome directed immune response instead of an anti-cancer directed immune response.

Besides the highly inflamed cluster 2, we identified three other clusters with a differential immune status that were found in every country. Cluster 1A, characterized by a moderate presence of T-, B- and mast cells; cluster 1B, presenting low number of immune cells; and cluster 1C, mainly dominated by macrophages and dendritic cells. Interestingly, both cluster 1A and 1C were enhanced for antigen presentation and showed higher expression of immune checkpoint related genes, such as *CD274* (PD-L1), *TIGIT*, *LAG3*, *IDO1* and *HAVCR* (TIM3), compared to cluster 1B. Thereby, these clusters likely contain tumors with a high likelihood responding to immune checkpoint inhibitors, while the opposite is true for the immune deserted cluster 1B. However, while cluster 1A has a moderate presence of T cells, which is well-known to response to immunotherapy [29], cluster 1C is enriched in macrophages and overexpressed the matrix remodeling process. It has been shown that tumor-associated macrophages (TAMs) limit the efficacy of immunotherapy. TAMS may reduce CD8+ T cell motility and confine them to the stroma and may contribute to ECM remodeling and disruption of its stiffness, thus limiting immune cell infiltration into the tumor and decreasing the therapeutic impact of ICIs [30–32]. Targeting macrophages could potentially improve immunotherapy response in these tumors [33]. On the other hand, cluster 1B is enriched for hypoxia, which is an important factor in immune excluded phenotypes [34]. In fact, increased intratumoral hypoxia and decreased CD8+ T cells have been shown in anti-PD-1 resistant head and neck tumors [35]. Hypoxia modulators hold promise to improve cancer immunotherapy, with ongoing efforts to develop highly selective hypoxia inhibitors [36].

As GCs from cluster 1A, 1B and 1C could be found in all countries, we concluded that both EU and LATAM countries have GCs with an immune composition that predisposes to a benefit from immune checkpoint inhibitors. Although Mexican GC patients in the highly inflamed cluster 2 contained a high number of T cells, these cancers lacked antigen presentation and cytotoxicity which argues in favor of an anti- microbiome immune response instead of an antitumor immune response. It is unknown whether these GCs will equally respond to ICIs compared to patients with cluster 1A tumors.

A growing number of studies have addressed the role of *H. pylori* infection on the efficacy of ICI therapy. While in preclinical data and in cancer types other than GC, *H. pylori* has been associated with lower effectiveness of anti-PD-1 immunotherapy [37], in the context of GC there have been contrasting results. In a retrospective analysis of a USA cohort of 215 metastatic GC patients treated with ICIs, those with a history of *H. pylori* infection (23%) had significantly shorter median progression-free survival (PFS) and overall survival (OS) [38]. In the largest retrospective study to date on the relationship between *H. pylori* and immunotherapy response, a positive relation was shown [39]. In a Chinese cohort of 636 MSS/EBV-negative GC patients who were treated with anti-PD-1/PD-L1 therapy, *H. pylori*-positive patients had significantly longer immune-related-PFS and -OS compared to *H. pylori*-negative [39]. Moreover, *H. pylori*-positive GCs had significantly higher densities of PD-L1+ and of non-exhausted CD8+ T cells in the TME, and shared molecular characteristics similar to those of immunotherapy-sensitive GC. These results are in line with our findings in Mexican GC patients presenting high-inflamed tumors.

We additionally found an association between high abundance of *Lactobacillus* sp. and immune cluster 2, which was common to EU and LATAM GCs [40]. While high relative abundance of *Lactobacillus* has been described in the stomach of GC patients in comparison to patients without GC, little is known about the immune profile associated to this genus [4, 5]. Interestingly, in a MSI-high colorectal cancer xenograft mouse model, infection with *Lactobacillus* synergized with anti-PD1 therapy by enhancing CD8+ T cells and reducing Foxp3+CD25+ Treg intratumoural cell infiltration [41]. Furthermore, in HER2-negative advanced gastric or GEJ adenocarcinoma patients, *Lactobacillus* was enriched in the gut microbiome of responders to anti-PD-1/PD-L1 therapy and was associated with better PFS [40].

Our study has several limitations, such as the lack of representation of certain areas from LATAM or EU, as well as limitations of the NanoString panel, which does not encompass all transcriptomic signaling pathways. Additionally, the quality of tissue samples in some cases was insufficient, preventing analysis in certain patients. On the other hand, the LEGACy project was designed to provide a comprehensive overview of the tumor characteristics in EU and LATAM patients, but did not incorporate functional assays. Nevertheless, altogether, this study from the LEGACy consortium showed that the immune microenvironment of both EU and LATAM countries are heterogeneous and that only a subgroup of GC has an inflamed immune infiltrate, likely requiring immunostimulatory drugs for reactivation. The fact that a subgroup of cancers, mainly composed by tumors from Mexico, are inflamed but not enriched for antigen presentation and cytotoxicity pathways, suggests an immune response against *H. pylori* rather than an active antitumor response. Further research is needed to determine whether both anti-microbiota and antitumor immune responses positively influence the efficacy of checkpoint inhibitors.

## Supplementary information

Supplementary Material

## Data Availability

The data presented in this article are available upon request to the corresponding author.

## References

[1] Bray F, Laversanne M, Sung H, Ferlay J, Siegel RL, Soerjomataram I, et al. Global cancer statistics 2022: GLOBOCAN estimates of incidence and mortality worldwide for 36 cancers in 185 countries. CA Cancer J Clin. 2024;74:229–63. 38572751 10.3322/caac.21834

[2] Global Cancer Observatory: Cancer Today. Lyon, France: International Agency for Research on Cancer; 2024. Available from: https://gco.iarc.who.int/today/en.

[3] de Martel C, Georges D, Bray F, Ferlay J, Clifford GM. Global burden of cancer attributable to infections in 2018: a worldwide incidence analysis. Lancet Glob Health. 2020;8:e180–e90. 31862245 10.1016/S2214-109X(19)30488-7

[4] Ferreira RM, Pereira-Marques J, Pinto-Ribeiro I, Costa JL, Carneiro F, Machado JC, et al. Gastric microbial community profiling reveals a dysbiotic cancer-associated microbiota. Gut. 2018;67:226–36. 29102920 10.1136/gutjnl-2017-314205 PMC5868293

[5] Mendes-Rocha M, Pereira-Marques J, Ferreira RM, Figueiredo C. Gastric Cancer: The Microbiome Beyond Helicobacter pylori. Curr Top Microbiol Immunol. 2023;444:157–84. 38231218 10.1007/978-3-031-47331-9_6

[6] Cancer of the Stomach - Cancer Stat Facts. 2024. Available from: https://seer.cancer.gov/statfacts/html/stomach.html.

[7] Lordick F, Carneiro F, Cascinu S, Fleitas T, Haustermans K, Piessen G, et al. Gastric cancer: ESMO Clinical Practice Guideline for diagnosis, treatment and follow-up. Ann Oncol. 2022;33:1005–20. 35914639 10.1016/j.annonc.2022.07.004

[8] Bang YJ, Van Cutsem E, Feyereislova A, Chung HC, Shen L, Sawaki A, et al. Trastuzumab in combination with chemotherapy versus chemotherapy alone for treatment of HER2-positive advanced gastric or gastro-oesophageal junction cancer (ToGA): a phase 3, open-label, randomised controlled trial. Lancet. 2010;376:687–97. 20728210 10.1016/S0140-6736(10)61121-X

[9] Kelly RJ, Ajani JA, Kuzdzal J, Zander T, Van Cutsem E, Piessen G, et al. Adjuvant Nivolumab in Resected Esophageal or Gastroesophageal Junction Cancer. N Engl J Med. 2021;384:1191–203. 33789008 10.1056/NEJMoa2032125

[10] Janjigian YY, Shitara K, Moehler M, Garrido M, Salman P, Shen L, et al. First-line nivolumab plus chemotherapy versus chemotherapy alone for advanced gastric, gastro-oesophageal junction, and oesophageal adenocarcinoma (CheckMate 649): a randomised, open-label, phase 3 trial. Lancet. 2021;398:27–40. 34102137 10.1016/S0140-6736(21)00797-2 PMC8436782

[11] Rha SY, Oh DY, Yañez P, Bai Y, Ryu MH, Lee J, et al. Pembrolizumab plus chemotherapy versus placebo plus chemotherapy for HER2-negative advanced gastric cancer (KEYNOTE-859): a multicentre, randomised, double-blind, phase 3 trial. Lancet Oncol. 2023;24:1181–95. 37875143 10.1016/S1470-2045(23)00515-6

[12] Janjigian YY, Kawazoe A, Bai Y, Xu J, Lonardi S, Metges JP, et al. Pembrolizumab plus trastuzumab and chemotherapy for HER2-positive gastric or gastro-oesophageal junction adenocarcinoma: interim analyses from the phase 3 KEYNOTE-811 randomised placebo-controlled trial. Lancet. 2023;402:2197–208. 37871604 10.1016/S0140-6736(23)02033-0

[13] Cabeza-Segura M, Gambardella V, Gimeno-Valiente F, Carbonell-Asins JA, Alarcón-Molero L, González-Vilanova A, et al. Integrative immune transcriptomic classification improves patient selection for precision immunotherapy in advanced gastro-oesophageal adenocarcinoma. Br J Cancer. 2022;127:2198–206. 36253523 10.1038/s41416-022-02005-z PMC9727124

[14] Lin SJ, Gagnon-Bartsch JA, Tan IB, Earle S, Ruff L, Pettinger K, et al. Signatures of tumour immunity distinguish Asian and non-Asian gastric adenocarcinomas. Gut. 2015;64:1721–31. 25385008 10.1136/gutjnl-2014-308252 PMC4680172

[15] Derks S, de Klerk LK, Xu X, Fleitas T, Liu KX, Liu Y, et al. Characterizing diversity in the tumor-immune microenvironment of distinct subclasses of gastroesophageal adenocarcinomas. Ann Oncol. 2020;31:1011–20. 32387455 10.1016/j.annonc.2020.04.011 PMC7690253

[16] Peng L, Qin BD, Xiao K, Xu S, Yang JS, Zang YS, et al. A meta-analysis comparing responses of Asian versus non-Asian cancer patients to PD-1 and PD-L1 inhibitor-based therapy. Oncoimmunology. 2020;9:1781333. 32923143 10.1080/2162402X.2020.1781333 PMC7458616

[17] van Schooten TS, Derks S, Jiménez-Martí E, Carneiro F, Figueiredo C, Ruiz E, et al. The LEGACy study: a European and Latin American consortium to identify risk factors and molecular phenotypes in gastric cancer to improve prevention strategies and personalized clinical decision making globally. BMC Cancer. 2022;22:646. 35692051 10.1186/s12885-022-09689-9 PMC9190072

[18] Siewert JR, Hölscher AH, Becker K, Gössner W. [Cardia cancer: attempt at a therapeutically relevant classification]. Chirurg. 1987;58:25–32. 3829805

[19] Martinez-Ciarpaglini C, Fleitas-Kanonnikoff T, Gambardella V, Llorca M, Mongort C, Mengual R, et al. Assessing molecular subtypes of gastric cancer: microsatellite unstable and Epstein-Barr virus subtypes. Methods for detection and clinical and pathological implications. ESMO Open. 2019;4:e000470. 31231566 10.1136/esmoopen-2018-000470 PMC6555614

[20] Bartley AN, Washington MK, Ventura CB, Ismaila N, Colasacco C, Benson AB, et al. HER2 Testing and Clinical Decision Making in Gastroesophageal Adenocarcinoma: Guideline From the College of American Pathologists, American Society for Clinical Pathology, and American Society of Clinical Oncology. Arch Pathol Lab Med. 2016;140:1345–63. 27841667 10.5858/arpa.2016-0331-CP

[21] Bankhead P, Loughrey MB, Fernández JA, Dombrowski Y, McArt DG, Dunne PD, et al. QuPath: Open source software for digital pathology image analysis. Sci Rep. 2017;7:16878. 29203879 10.1038/s41598-017-17204-5 PMC5715110

[22] Pinto-Ribeiro I, Ferreira RM, Pereira-Marques J, Pinto V, Macedo G, Carneiro F, et al. Evaluation of the Use of Formalin-Fixed and Paraffin-Embedded Archive Gastric Tissues for Microbiota Characterization Using Next-Generation Sequencing. Int J Mol Sci. 2020;21:1096. 32046034 10.3390/ijms21031096 PMC7037826

[23] Edgar R. UNOISE2: improved error-correction for Illumina 16S and ITS amplicon sequencing. bioRxiv; 2016, https://www.biorxiv.org/content/10.1101/081257v1.

[24] Edgar R. SINTAX: a simple non-Bayesian taxonomy classifier for 16S and ITS sequences. bioRxiv; 2016, https://www.biorxiv.org/content/10.1101/074161v1.

[25] Edgar RC. UPARSE: highly accurate OTU sequences from microbial amplicon reads. Nat Methods. 2013;10:996–8. 23955772 10.1038/nmeth.2604

[26] Benjamini Y, Hochberg Y. Controlling the False Discovery Rate: A Practical and Powerful Approach to Multiple Testing. J R Stat Soc Ser B. 2018;57:289–300.

[27] Allison CC, Kufer TA, Kremmer E, Kaparakis M, Ferrero RL. Helicobacter pylori induces MAPK phosphorylation and AP-1 activation via a NOD1-dependent mechanism. J Immunol. 2009;183:8099–109. 20007577 10.4049/jimmunol.0900664

[28] He J, Nascakova Z, Leary P, Papa G, Valenta T, Basler K, et al. Inactivation of the tumor suppressor gene Apc synergizes with H. pylori to induce DNA damage in murine gastric stem and progenitor cells. Sci Adv. 2023;9:eadh0322. 37967175 10.1126/sciadv.adh0322 PMC10651120

[29] Raskov H, Orhan A, Christensen JP, Gögenur I. Cytotoxic CD8(+) T cells in cancer and cancer immunotherapy. Br J Cancer. 2021;124:359–67. 32929195 10.1038/s41416-020-01048-4 PMC7853123

[30] Peranzoni E, Lemoine J, Vimeux L, Feuillet V, Barrin S, Kantari-Mimoun C, et al. Macrophages impede CD8 T cells from reaching tumor cells and limit the efficacy of anti-PD-1 treatment. Proc Natl Acad Sci USA. 2018;115:E4041–e50. 29632196 10.1073/pnas.1720948115 PMC5924916

[31] Mai Z, Lin Y, Lin P, Zhao X, Cui L. Modulating extracellular matrix stiffness: a strategic approach to boost cancer immunotherapy. Cell Death Dis. 2024;15:307. 38693104 10.1038/s41419-024-06697-4 PMC11063215

[32] Yu K-X, Yuan W-J, Wang H-Z, Li Y-X. Extracellular matrix stiffness and tumor-associated macrophage polarization: new fields affecting immune exclusion. Cancer Immunol, Immunother. 2024;73:115. 38693304 10.1007/s00262-024-03675-9 PMC11063025

[33] Duan Z, Luo Y. Targeting macrophages in cancer immunotherapy. Signal Transduct Target Ther. 2021;6:127. 33767177 10.1038/s41392-021-00506-6 PMC7994399

[34] Pietrobon V, Marincola FM. Hypoxia and the phenomenon of immune exclusion. J Transl Med. 2021;19:9. 33407613 10.1186/s12967-020-02667-4 PMC7788724

[35] Zandberg DP, Menk AV, Velez M, Normolle D, DePeaux K, Liu A, et al. Tumor hypoxia is associated with resistance to PD-1 blockade in squamous cell carcinoma of the head and neck. J Immunother Cancer. 2021;9:e002088. 33986123 10.1136/jitc-2020-002088 PMC8126285

[36] Janji B, Chouaib S. The Promise of Targeting Hypoxia to Improve Cancer Immunotherapy: Mirage or Reality? Front Immunol. 2022;13:880810. 35795658 10.3389/fimmu.2022.880810 PMC9251545

[37] Oster P, Vaillant L, Riva E, McMillan B, Begka C, Truntzer C, et al. Helicobacter pylori infection has a detrimental impact on the efficacy of cancer immunotherapies. Gut. 2022;71:457–66. 34253574 10.1136/gutjnl-2020-323392 PMC8862014

[38] Magahis PT, Maron SB, Cowzer D, King S, Schattner M, Janjigian Y, et al. Impact of Helicobacter pylori infection status on outcomes among patients with advanced gastric cancer treated with immune checkpoint inhibitors. J Immunother Cancer. 2023;11:e007699. 37899129 10.1136/jitc-2023-007699 PMC10619027

[39] Jia K, Chen Y, Xie Y, Wang X, Hu Y, Sun Y, et al. Helicobacter pylori and immunotherapy for gastrointestinal cancer. Innovation. 2024;5:100561. 38379784 10.1016/j.xinn.2023.100561 PMC10878118

[40] Han Z, Cheng S, Dai D, Kou Y, Zhang X, Li F, et al. The gut microbiome affects response of treatments in HER2-negative advanced gastric cancer. Clin Transl Med. 2023;13:e1312. 37381590 10.1002/ctm2.1312 PMC10307992

[41] Fong W, Li Q, Ji F, Liang W, Lau HCH, Kang X, et al. Lactobacillus gallinarum-derived metabolites boost anti-PD1 efficacy in colorectal cancer by inhibiting regulatory T cells through modulating IDO1/Kyn/AHR axis. Gut. 2023;72:2272–85. 37770127 10.1136/gutjnl-2023-329543 PMC10715476

